# Silver Nanoparticles as a Novel Tissue Preservative: A Comparative Study with 10% Neutral Buffered Formalin

**DOI:** 10.3390/ijms26115335

**Published:** 2025-06-01

**Authors:** Safa Taha, Amina Ismaeel, Muna Aljishi, Samvel Selvam, Angeleena Esther, Khaled Greish

**Affiliations:** 1 Department of Molecular Medicine, College of Medicine and Health Sciences, Princess Al Jawhara Center for Molecular Medicine, Genetics and Inherited Diseases, Arabian Gulf University, Manama 329, Bahrain; safat@agu.edu.bh (S.T.); munajma@agu.edu.bh (M.A.); 2 Department of Pathology, College of Medicine and Health Sciences, Arabian Gulf University, Manama 329, Bahrain; aminaaya@agu.edu.bh (A.I.); samvels@agu.edu.bh (S.S.); 3 Clinical Research Center, College of Medicine and Health Sciences, Arabian Gulf University, Manama 329, Bahrain; angeleenae@agu.edu.bh

**Keywords:** silver nanoparticles (AgNPs), BALB/c mice, 10% neutral buffered formalin (10% NBF), GAPDH, nucleic acid extraction, protein extraction, Western blot, RT-PCR

## Abstract

Tissue preservation plays an essential role in biomedical research and histopathological applications. Traditional methods, despite their efficiency, are associated with compromised long-term tissue integrity and probable ecotoxicities. This study explores the application of silver nanoparticles (AgNPs), known for their antimicrobial properties, as a potential tissue preservative. In this work, AgNPs were synthesized via a chemical reduction method. Heart, liver, and kidney tissues were obtained from BALB/c mice and preserved using 10% neutral buffered formalin (NBF) and AgNPs solution for 72 h. Preservation efficiency was assessed by quantifying and measuring DNA and RNA integrity, evaluating protein stability, and conducting histopathological examinations. This study aimed to compare the performance of AgNPs against 10% NBF across these parameters to determine their suitability as an alternative fixative. Our results showed that AgNPs solution maintained consistent DNA, RNA, and protein concentrations/quality across all tissues over 72 h, whereas formalin treatment led to degradation over time. Conversely, 10% NBF demonstrated better preservation of tissue morphology. These results highlighted the differential strengths of each fixative, with AgNPs excelling in molecular preservation and NBF in structural integrity. Overall, AgNPs exhibited superior qualitative and quantitative preservation of nucleic acids and intracellular proteins, indicating their potential as an alternative to formalin for molecular testing. Despite their demonstrated efficacy in biomolecular preservation, further studies are needed to optimize tissue morphology preservation.

## 1. Introduction

Tissue preservation and fixation play a vital role in research and have significant implications in medicine, particularly in transplantation, regenerative medicine, forensics, diagnostic pathology, and surgery. Choosing the proper tissue fixative significantly impacts the quality of tissue sections, tissue morphology, protein integrity, and the quality of downstream molecular analyses [1]. Historically, formalin, an aqueous solution of formaldehyde, has been widely employed as a gold standard for tissue preservation due to its efficacy and availability. However, formalin has been classified as a type 1 carcinogen, posing significant health risks to laboratory personnel and environmental hazards due to its toxicity and carcinogenic properties [2,3]. Another significant challenge associated with formalin use is respiratory and mucosal irritation in both clinical and laboratory settings, which can lead to upper respiratory illnesses characterized by symptoms such as cough, dyspnea, and bronchospasm [4]. It can also lead to corrosive injuries to the skin, leading to burns and irritation [5]. Studies on mice have shown that formalin exposure can cause significant damage to vital organs such as the liver and kidneys, including cell deformity, tissue degeneration, and hemorrhages [6,7]. Additionally, formalin fixation leads to significant degradation of nucleic acids due to slow fixation and enzymatic activity, affecting the quality of molecular analyses [8,9]. Moreover, the handling and disposal of formalin also incur legal and environmental costs, prompting the need for safer alternatives [2,3].

Several alternatives to formalin have been explored, including ethanol-based fixatives, glyoxal, and vacuum-based tissue transfer methods, which aim to reduce toxicity while maintaining preservation quality [3,9]. However, these alternatives often compromise either morphological or molecular integrity. Nanotechnology offers novel solutions, with silver nanoparticles (AgNPs) gaining attention for their potent antimicrobial properties and low toxicity at controlled concentrations [10,11]. AgNPs penetrate bacterial cell walls, disrupt membranes, and induce cell death, ensuring sterility in preserved tissues [11,12]. Their physicochemical properties, such as size and surface chemistry, enhance their efficacy [13]. This study aims to evaluate the efficacy of AgNPs in preserving tissue integrity at the molecular and morphological levels, benchmarking their performance against the gold standard, formalin fixation, to assess their potential as a safer, effective alternative.

## 2. Results

### 2.1. Silver Synthesis and Characterization

**Figure 1:**
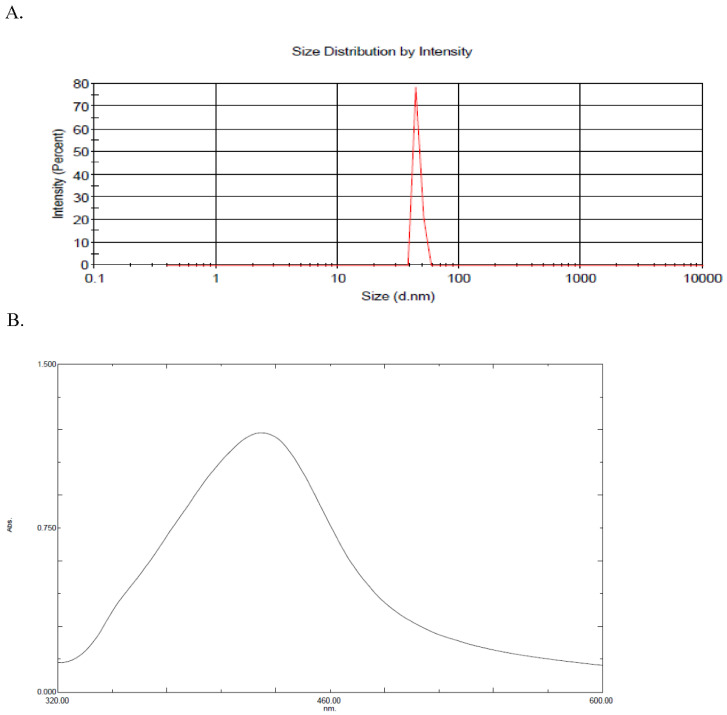
(**A**,**B**): The average size was 45.3 nm, as measured by DLS, with a single peak at 424 nm.

The AgNPs solution was prepared and concentrated as detailed in Section 4.1. The resultant particles showed a maximum visible light absorbance at 424 nm as measured by UV-2600 (Shimadzu-Japan, Kyoto, Japan). The AgNPs showed an average size of 45.3 nm, as measured by Malvern ZEN3600 Zeta Sizer Nano series (Malvern Instruments Inc., Westborough, MA, USA). The prepared nanoparticles had a concentration of 20 μg/mL. AgNPs were further concentrated in a heating oven (50 °C) to acquire the operational concentration in this study of 50 μg/mL, as illustrated in Figure 1.

### 2.2. DNA Concentration and Purity

**Figure 2:**
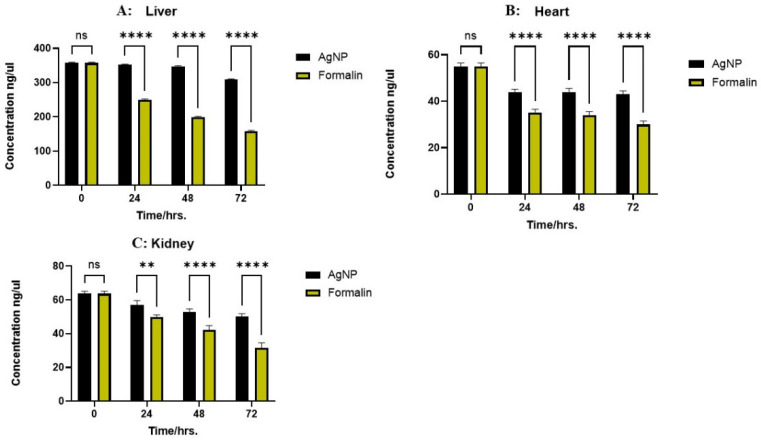
Concentration of extracted DNA (ng/µL) from the liver, kidneys, and heart at different time intervals (0, 24, 48, and 72 h). The bars represent the two preservative treatments: silver nanoparticles (AgNPs) and 10% neutral buffered formalin. The data represent the mean of three biological replicates (n = 3) measured in parallel for each tissue type and time point. Statistical significance between treatments is indicated as follows: (**A**) Liver: No significant difference (ns) is observed at 0 h. Significant differences are observed at 24, 48, and 72 h (*p* < 0.0001 for all time points, indicated by ****). (**B**) Heart: No significant difference (ns) is observed at 0 h. Significant differences are observed at 24, 48, and 72 h (*p* < 0.0001 for all time points, indicated by ****). (**C**) Kidney: No significant difference (ns) is observed at 0 h. Significant differences are observed at 24 h (*p* < 0.01, indicated by **) and at 48 and 72 h (*p* < 0.0001, indicated by ****).

In the liver, the saline control exhibited an initial DNA concentration of 357.00 ± 2.646 ng/μL. AgNPs treatment resulted in a gradual decrease, from 352.00 ± 2.000 ng/μL at 24 h to 309.33 ± 1.528 ng/μL at 72 h, while 10% NBF induced a more pronounced decline, from 250.00 ± 2.000 ng/μL at 24 h to 157.67 ± 2.517 ng/μL at 72 h. These differences were statistically significant at 24, 48, and 72 h (*p* < 0.0001). In the kidney tissue samples, the saline control showed 63.67 ± 1.528 ng/μL at 0 h. AgNPs treatment led to a gradual decrease, from 57.00 ± 2.646 ng/μL at 24 h to 50.33 ± 1.528 ng/μL at 72 h, while 10% NBF showed a more significant decrease, from 49.67 ± 1.528 ng/μL at 24 h to 31.67 ± 2.887 ng/μL at 72 h. Statistical significance was observed at 24 h (*p* < 0.01) and 48 and 72 h (*p* < 0.0001). In the heart, the saline control showed 55.00 ± 1.528 ng/μL at 0 h. AgNPs treatment resulted in a minimal decrease, from 44.00 ± 1.155 ng/μL at 24 h to 43.00 ± 1.528 ng/μL at 72 h, while 10% NBF also showed a decrease, from 35.00 ± 1.528 ng/μL at 24 h to 30.00 ± 1.528 ng/μL at 72 h. These differences were statistically significant at 24, 48, and 72 h (*p* < 0.0001). As illustrated in Figure 2, overall, tissues preserved in 10% NBF consistently showed a substantial reduction in DNA concentration across all tissue types compared to AgNPs solution, indicating greater DNA degradation. This was reflected in the highly significant *p*-values observed, suggesting that the AgNPs solution is comparatively better at DNA preservation over 72 h.

### 2.3. Effect of Preservation Time on Nucleic Acid and Protein Integrity

Initial assessments at 0 h confirmed high-quality DNA, RNA, and protein extraction across all samples, establishing a baseline for evaluating preservation efficacy over time. The data demonstrate that AgNPs-preserved tissues maintained nucleic acid and protein integrity more effectively than formalin-preserved tissues after 24, 48, and 72 h.

**Figure 3:**
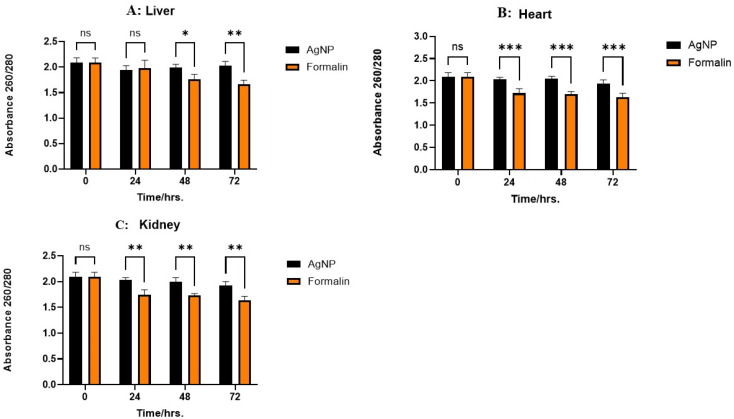
Absorbance ratio (260/280) for liver, heart, and kidney tissues (**A**–**C**) over time (0, 24, 48, and 72 h) under different preservative conditions (saline, AgNP, and 10% formalin). The data represent the mean of three biological replicates (n = 3) measured in parallel for each tissue type and time point. * *p* < 0.05; ** *p* < 0.01; *** *p* < 0.001. “ns”: not significant.

DNA purity was measured using the absorbance ratio at 260 nm and 280 nm. Figure 3 illustrates the absorbance ratio from liver, kidney, and heart tissues preserved using either AgNPs or 10% NBF at 0, 24, 48, and 72 h. In the liver, no significant differences in DNA purity were observed between the two preservation methods at 0 and 24 h. However, AgNPs-preserved samples exhibited significantly higher purity at 48 h (*p* < 0.05) and 72 h (*p* < 0.01). For kidney and heart tissues, while no differences were observed at 0 h, AgNP preservation resulted in significantly higher DNA purity at 24, 48, and 72 h (*p* < 0.01 for kidney; *p* < 0.001 for heart). These findings suggest that AgNPs are superior to 10% NBF for maintaining DNA purity, particularly over extended periods, with varying degrees of effectiveness across different tissues.

**Figure 4:**
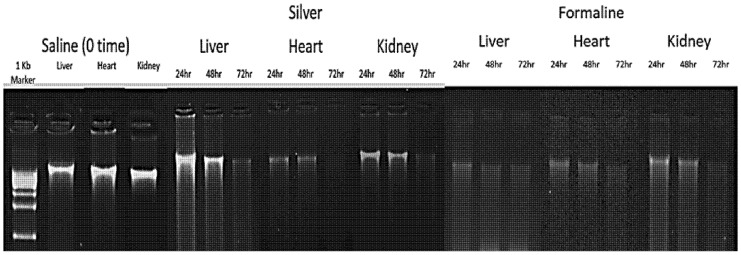
DNA extracted from liver, heart, and kidney samples preserved in saline (0 time), formalin, and AgNPs using 0.6% agarose gel electrophoresis. AgNPs-preserved samples displayed better DNA preservation compared to formalin, with clearer bands at 24 and 48 h, though some degradation was evident by 72 h, particularly in kidney samples.

Moreover, the integrity of DNA extracted from liver, heart, and kidney samples preserved in saline (0 time), formalin, and AgNPs was also evaluated using 0.6% agarose gel electrophoresis, as described in Section 4.3.3. Figure 4 illustrates the results, visualized on the gel, revealing distinct banding patterns across the samples. DNA from saline-treated samples (0 time) showed intact, high-molecular-weight bands for all tissues, indicating minimal degradation. In contrast, formalin-preserved samples exhibited significant DNA degradation, with smeared bands observed at 24, 48, and 72 h across all tissues, suggesting that formalin compromises DNA integrity over time. AgNPs-preserved samples displayed better DNA preservation compared to formalin, with clearer bands at 24 and 48 h, though some degradation was evident by 72 h, particularly in kidney samples. These findings highlight AgNPs as a more effective preservative for maintaining DNA integrity than formalin, while saline (0 time) remains the baseline for optimal DNA quality.

### 2.4. Preserving RNA Concentration and Quality: Tissue-Specific Responses to Different Treatments

**Figure 5:**
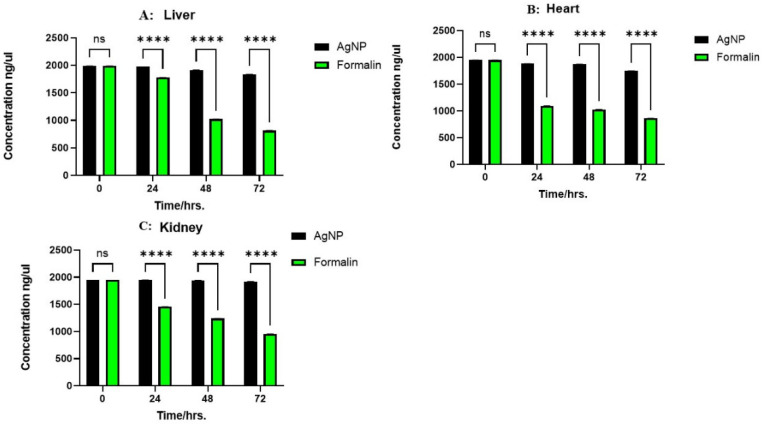
Concentration of extracted RNA (ng/µL) in the liver, heart, and kidney (**A**–**C**) at different time intervals (0, 24, 48, and 72 h). The bars represent preservative treatments, AgNPs and 10% NBF, with statistical significance between groups indicated by **** (*p* < 0.0001). “ns”: not significant. The data represent the mean of three biological replicates (n = 3) measured in parallel for each tissue type and time point.

The effects of the three preservation methods on RNA concentrations across the tissues over a 72-h period were examined. Saline consistently maintained stable RNA concentrations across all three tissues throughout this study (1991.30 ± 1.310 ng/μL). AgNPs treatment resulted in a gradual, tissue-dependent decline in RNA levels while, in contrast, 10% NBF induced a significant and rapid reduction in RNA concentration across all tissues, with the most pronounced decrease observed in the heart, from 1097 ± 2.310 ng/μL at 24 h to a mere 864 ± 2.012 ng/μL at the 72-h mark (*p* < 0.001). The consistent stability observed with saline, the moderate decline with AgNPs, and the substantial reductions with 10% NBF are clearly visualized in Figure 5. These findings suggest that preservation with 10% NBF has a significantly detrimental impact on RNA concentration over time compared to the AgNPs solution. Thus, these insights can inform the selection of appropriate preservative strategies for various tissue types, depending on the specific research needs and the desired preservation outcomes.

**Figure 6:**
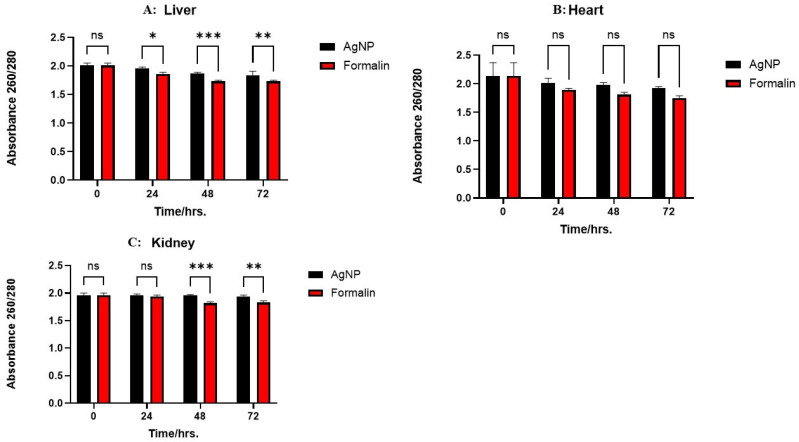
Absorbance ratio (260/280) for liver, heart, and kidney tissues (**A**–**C**) over time (0, 24, 48, and 72 h). The bars represent preservative treatments, AgNPs and 10% NBF, with statistical significance between groups indicated as follows: Liver (**A**): No significant difference (ns) is observed at 0 h. Significant differences are observed at 24 h (*p* < 0.05, indicated by *), 48 h (*p* < 0.001, indicated by ***), and 72 h (*p* < 0.01, indicated by **). Kidney (**C**): No significant difference (ns) is observed at 0 and 24 h. Significant differences are observed at 48 h (*p* < 0.001, indicated by ***) and 72 h (*p* < 0.01, indicated by **). The data represent the mean of three biological replicates (n = 3) measured in parallel for each tissue type and time point.

The relationships between various tissue types and the impact of different preservative treatments on the quality of RNA nucleic acids extracted were evaluated by analyzing the absorbance ratios at 260 nm and 280 nm. In the liver, AgNPs treatment resulted in a slight absorbance ratio decline from 1.96 to 1.84 over 72 h, while 10% NBF showed a more substantial decrease from 1.86 to 1.73. In the kidney, AgNPs maintained a stable ratio of around 1.95, whereas 10% NBF decreased from 1.94 to 1.83. Similarly, in the heart, the AgNPs ratio remained stable at around 2.0, while the 10% NBF declined from 1.89 to 1.75. Thus, 10% NBF consistently demonstrated a more significant reduction in absorbance ratios, indicating a greater impact on nucleic acid quality compared to AgNPs across all tested tissues. As clearly illustrated in Figure 6, the AgNPs treatment consistently exhibited relatively stable absorbance ratios across all tissues, while the 10% NBF treatment resulted in more pronounced declines. These findings suggest that AgNPs effectively maintained nucleic acid quality. Conversely, 10% NBF demonstrated a detrimental effect, significantly reducing absorbance ratios, particularly in the heart and liver tissues, indicating a greater impact on nucleic acid degradation.

### 2.5. GAPDH Expression Determination in Multiple Preservatives

**Figure 7:**
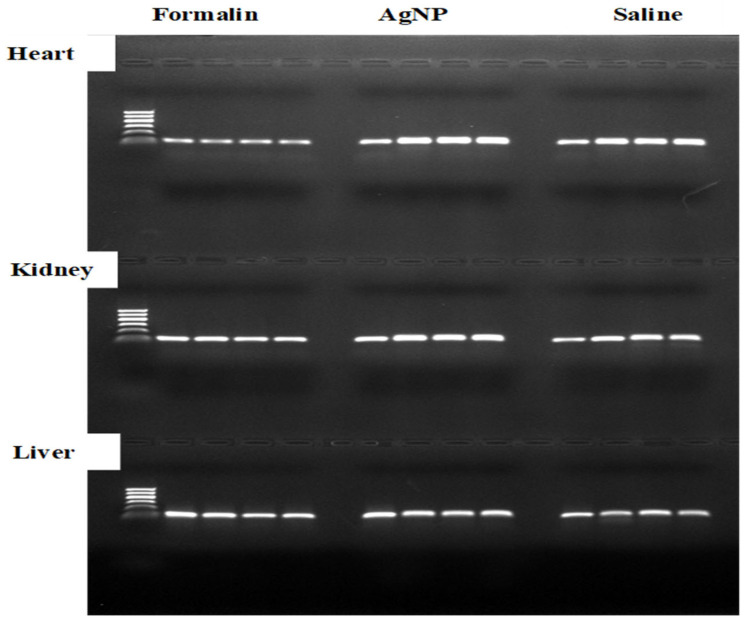
RT-PCR analysis of GAPDH expression across all tissues preserved in the different preservatives. The samples were evaluated at intervals of 0, 24, 48, and 72 h. The bands represent the relative expression levels of GAPDH under different conditions.

GAPDH, a housekeeping gene, is commonly used as a control gene for normalizing gene expression data [14]. Significant changes in the gene expression levels across different preservatives and time periods were identified by a RT-PCR study of GAPDH expression. The samples maintained in AgNPs demonstrated the highest expression levels of GAPDH across all time points (0, 24, 48, and 72 h). This indicates that AgNPs are effective in preserving the integrity and expression of this housekeeping gene during storage. The samples preserved in saline exhibited significant expression levels of GAPDH, akin to the AgNPs group. The expression levels in the saline group were comparable to, and, in some instances, slightly elevated compared to, the AgNPs group, suggesting that saline is equally effective in maintaining GAPDH expression. Although lower than the other two preservatives, the 10% NBF group exhibited relatively stable expression of the housekeeping gene throughout the assessed time intervals. These findings indicate that both AgNPs and saline are more effective than 10% NBF in maintaining GAPDH expression. Figure 7 depicts the RT-PCR analysis of GAPDH expression across all tissues over a 72-h period.

**Figure 8:**
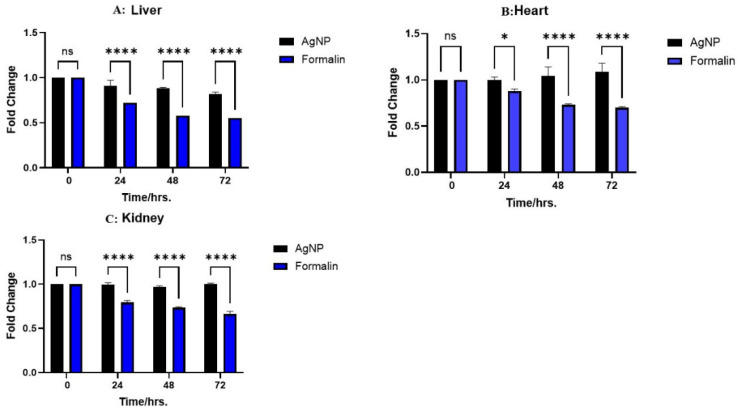
Expression levels of the housekeeping gene GAPDH in the three different tissue types (**A**–**C**) under 10% NBF and AgNPs preservation treatments and time intervals. Fold change values are shown for AgNPs and 10% NBF. The data represent the mean of three biological replicates (n = 3) measured in parallel for each tissue type and time point. Statistical significance between treatments is indicated as follows: Liver (**A**): No significant difference (ns) is observed at 0 h. Significant differences are observed at 24, 48, and 72 h (*p* < 0.0001 for all time points, indicated by ****). Heart (**B**): No significant difference (ns) is observed at 0 h. Significant differences are observed at 24 h (*p* < 0.05, indicated by *) and at 48 and 72 h (*p* < 0.0001 for both time points, indicated by ****). Kidney (**C**): No significant difference (ns) is observed at 0 h. Significant differences are observed at 24, 48, and 72 h (*p* < 0.0001 for all time points, indicated by ****).

GAPDH band intensity was analyzed using the AzurSpot software version 14.2. The heart tissue stored in saline at 0 h demonstrated the highest GAPDH expression level, approximately 1.2 on the intensity scale. The heart tissue stored in AgNPs exhibited consistently high GAPDH expression levels, ranging from approximately 0.9 to 1.0, at the 24-, 48-, and 72-h time points, while heart tissues preserved in 10% NBF showed a gradual decline in GAPDH expression over time, starting at approximately 0.7 at 24 h and decreasing to nearly 0.4 by 72 h. Similarly, kidney tissue treated with saline at 0 h exhibited the highest GAPDH expression level, surpassing 1.1 on the intensity scale. This indicated the peak expression observed in all kidney samples. Kidney samples preserved in silver at 24, 48, and 72 h displayed intensity values between approximately 0.9 and 1.0 on the intensity scale, indicating that AgNPs effectively maintained strong GAPDH expression in kidney samples. The sustained increase in GAPDH levels in kidney tissue during the silver treatment periods is noteworthy, especially compared to the gradual decrease observed in the 10% NBF-preserved kidney samples over the same duration, starting at approximately 0.6 at 24 h and decreasing to about 0.4 by 72 h. The liver tissue stored in saline at 0 h demonstrated the highest GAPDH expression level, surpassing 1.0 on the intensity scale. Liver tissue preserved in AgNPs solution demonstrated consistently high GAPDH expression levels, ranging from approximately 0.8 to 0.9 at the 24, 48, and 72-h time points. In contrast, liver tissue preserved in 10% NBF showed a notable decrease in GAPDH expression over time, starting at approximately 0.6 at 24 h and declining to about 0.3 by 72 h. These findings, illustrated in Figure 8, indicate that saline- and AgNP-based preservation methods were more effective in maintaining GAPDH expression levels than 10% NBF across all three tissue types and time points.

### 2.6. Protein Extraction and Analysis

**Figure A1:**
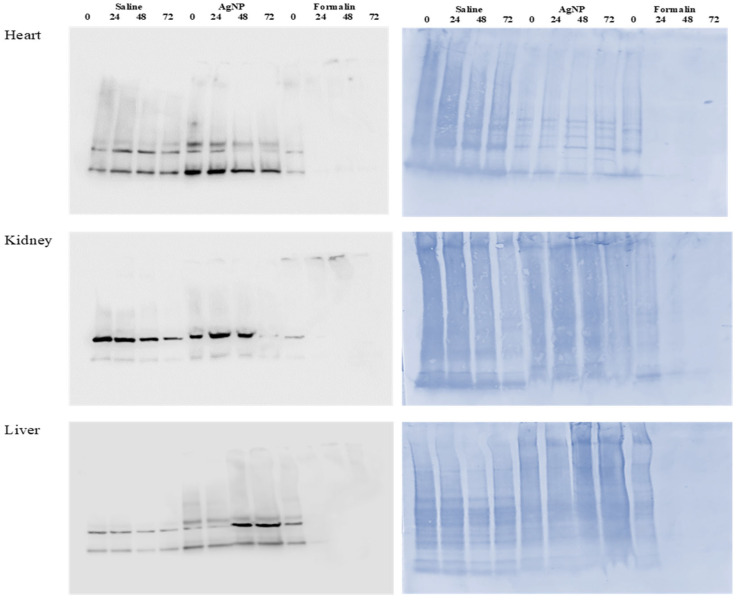
The image displays the results of a Western blot test that used an antibody to find the GAPDH protein in heart, kidney, and liver samples. The Western blot showed clear GAPDH protein bands in the organ samples, showing that this important protein was expressed consistently. The strength and clarity of the GAPDH bands changed based on the preservation method applied. The GAPDH bands in the samples preserved with AgNPs were clear and strong in the heart and kidney. This suggests that this preservation method is the best at keeping the structure and responsiveness of GAPDH intact. The GAPDH bands in the samples treated with 10% neutral buffered formalin (10% NBF) looked a bit less clear. This suggests that these preservation methods may have affected the GAPDH protein structure or how well the antibody could recognize it.

The protein profiles of the preserved organs were examined using SDS-PAGE and Western blot analysis. AgNPs and saline-treated tissues yielded clearer, better-resolved protein bands compared to 10% NBF, indicating superior protein preservation. AgNPs-treated tissues maintained protein integrity and GAPDH immunoreactivity over time more effectively than 10% NBF. Consistent GAPDH detection across all treatments validated its use as a reliable loading control and reference protein for comparative protein analysis. These results highlight AgNPs’ potential for improved protein preservation, which may benefit downstream proteomic and biomarker discovery studies. Appendix A Figure A1 demonstrates that protein bands from AgNPs-preserved organ samples exhibited greater distinctness and resolution compared to those from 10% NBF-preserved samples.

### 2.7. Histopathologic Evaluation of Tissue Samples

**Figure 9:**
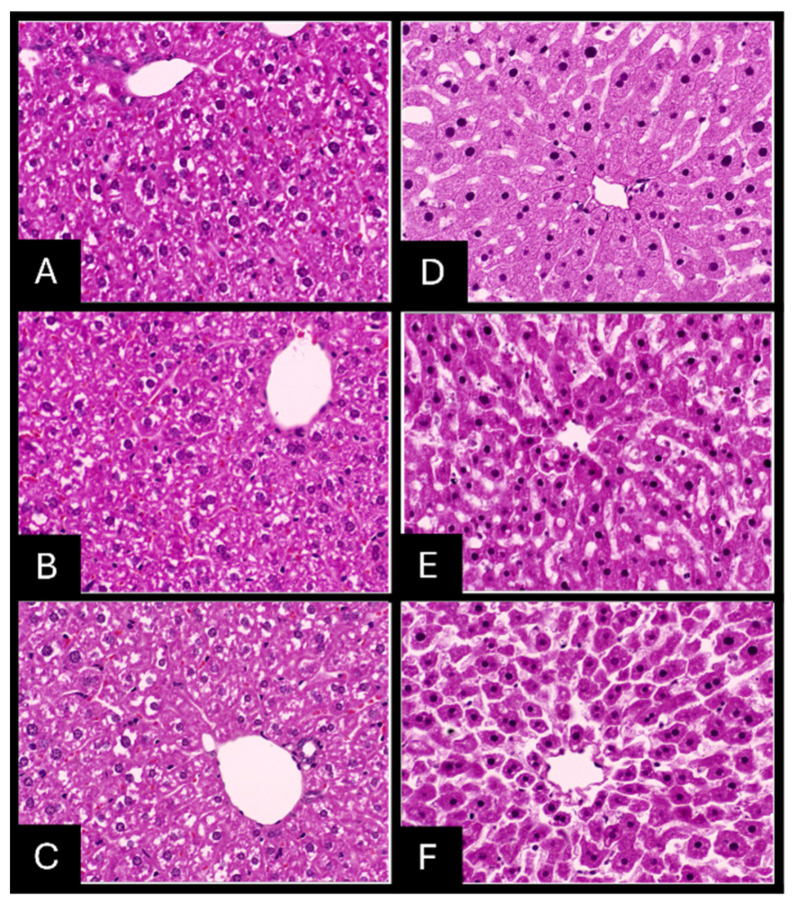
H & E staining of liver (40×). Tissue fixed with 10% NBF (**left panel**) or AgNP solution (**right panel**) for 24 h (**A**,**D**), 48 h (**B**,**E**), and 72 h (**C**,**F**).

**Figure 10:**
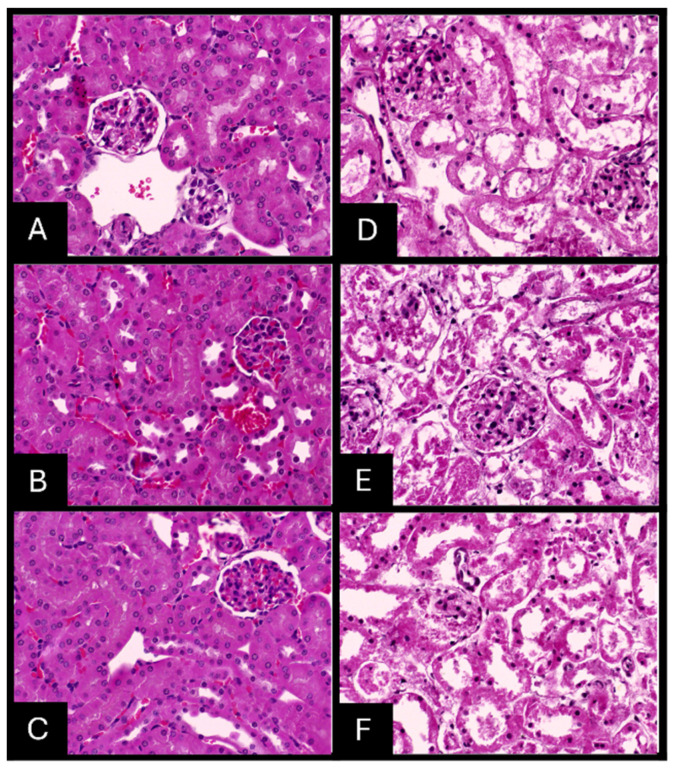
H & E staining of the kidney (40×). Tissue fixed with 10% NBF (**left panel**) or AgNP solution (**right panel**) for 24 h (**A**,**D**), 48 h (**B**,**E**) and 72 h (**C**,**F**).

**Figure 11:**
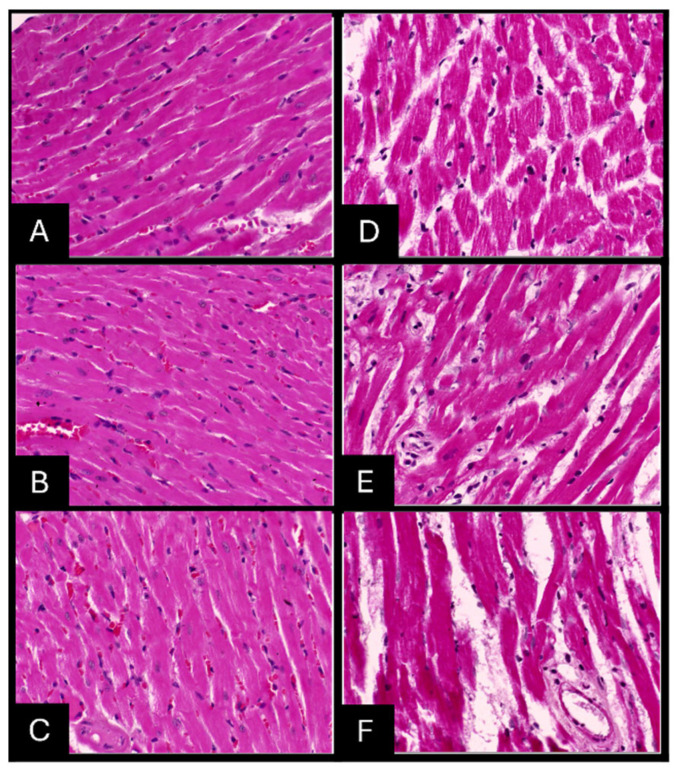
H & E staining of the heart (40×). Tissue fixed with 10% NBF (**left panel**) or AgNP solution (**right panel**) for 24 h (**A**,**D**), 48 h (**B**,**E**) and 72 h (**C**,**F**).

Gross examination of AgNPs-fixed specimens revealed a softer and paler texture and increased difficulty in microtome sectioning compared to 10% NBF-fixed tissues. Microscopically, AgNPs-fixed paraffin-embedded tissue stained with H&E staining showed pyknotic nuclei, shrinkage of cells, poor cytologic features, blurred disintegrated cytoplasmic membrane contour, and granular cytoplasm with disintegration of tissue morphology (Figure 9, Figure 10 and Figure 11).

The histopathologic scoring of tissue morphology was calculated and compared to the 10% NBF-fixed paraffin-embedded tissue stained with H&E (Appendix A; Table A1). Scores were found to be inadequate for tissues fixed with an AgNPs solution, in contrast to tissues fixed with 10% NBF, which were adequately preserved at the three time points of fixation for the three organs tested, indicating that AgNPs did not effectively preserve tissue morphology compared to the established 10% NBF standard.

## 3. Discussion

The application of AgNPs as a tissue preservative remains a relatively under-explored area of research. While numerous studies have investigated the antimicrobial properties of AgNPs and their applications in biomedicine, a notable shortage of research remains in specifically exploring their preservative capabilities in tissues. Consequently, the current investigation aims to elucidate this process, with the findings from this study providing significant insights into the preservation capabilities and limitations of AgNPs compared to the gold standard, formalin.

Our research indicated that AgNPs provided excellent preservation of DNA, RNA, and protein integrity and quantity across all tissue types over a 72-h timeframe, a notable improvement relative to formalin fixation. These results are consistent with the widely documented degradation of nucleic acids by formalin, resulting in a reduced quality of extracted molecules and a subsequent negative impact on sensitive molecular analyses [15,16]. An earlier study on biosynthesized AgNPs for preserving mulberry leaves demonstrated their superior performance in preventing microbial proliferation and maintaining structural integrity [17], suggesting that similar principles may apply to biological tissues. Further research has demonstrated the inhibitory effect of AgNPs on voltage-gated sodium channels [18,19], which may contribute to their preservative effects by stabilizing cellular functions. Another contributing mechanism to the molecular preservation observed with AgNPs was reported by Ekaterina O [20], who demonstrated that AgNPs induce the inactivation of sulfur- and phosphorus-rich enzymes, thereby disrupting cellular metabolism. This contrasts with formalin, which slowly inactivates intracellular enzymes, allowing time for biomolecular degradation by endogenous tissue enzymes. However, AgNPs exhibited suboptimal morphological preservation compared to formalin, as detailed in the limitations.

Compared to formalin, AgNPs offer reduced toxicity and superior biomolecular preservation, which are critical for molecular analyses. However, their suboptimal morphological preservation limits histopathological applications. Alternative fixatives, such as ethanol or glyoxal, also struggle to balance molecular and morphological preservation [9]. Optimizing AgNPs formulations could address these limitations, leveraging their antimicrobial and low-toxicity properties [13]. The size of the AgNPs is likely a critical factor influencing tissue preservation effectiveness, as smaller nanoparticles may penetrate tissues more effectively, enhancing antimicrobial and enzyme-inactivating properties, while larger particles could improve structural stabilization but potentially reduce tissue permeability. Although our study used a standardized AgNP size, variations in nanoparticle size could modulate preservation outcomes, as smaller AgNPs have been shown to exhibit more substantial antimicrobial effects in other applications [21]. Further studies investigating the impact of AgNP size on both molecular and morphological preservation are needed to optimize their utility as a tissue preservative.

Safety concerns associated with formalin further highlight the importance of exploring alternatives like AgNPs for tissue preservation. Formalin’s classification as a type 1 carcinogen is associated with significant health risks, including short-term respiratory, nasopharyngeal, ocular, and dermal irritation, as well as long-term metaplastic changes and potential carcinogenesis in the naso- and oropharynx [4,22]. In contrast, AgNPs offer multiple advantages beyond reduced toxicity when used at controlled concentrations. These include superior preservation of DNA, RNA, and protein integrity, as demonstrated in our study (Section 2.2, Section 2.3 and Section 2.4), which is critical for downstream molecular analyses such as PCR and Western blotting, where formalin’s degradative effects are a known limitation [8,15,16]. Additionally, AgNPs’ broad-spectrum antimicrobial activity prevents microbial proliferation, ensuring tissue sterility without additional preservatives [10,11,12]. Their ability to inactivate sulfur- and phosphorus-rich enzymes further supports biomolecular preservation by halting endogenous degradation processes [19]. While reduced toxicity is a significant driver for adopting AgNPs, these multifaceted benefits—biomolecular preservation, antimicrobial efficacy, and enzyme inactivation—position AgNPs as a promising alternative to formalin. However, challenges in morphological preservation, as outlined in the limitations section, indicate that further optimization is needed to achieve a balance between adequate morphological and superior biomolecular preservation. This could involve modifying AgNPs formulations or combining them with other fixatives to enhance histological outcomes. Future studies optimizing these aspects, as outlined in the final paragraph, could further enhance AgNPs’ potential.

The potential of AgNPs as a tissue preservative is further supported by their growing applications in biomedical fields, such as drug delivery, wound healing, and anti-nociceptive therapies [10,18]. These applications underscore AgNPs’ versatility and their capacity to address the limitations of traditional fixatives like formalin. Future studies should focus on optimizing AgNPs-based preservation protocols, including concentration gradients and delivery methods, to improve tissue morphology while maintaining their superior biomolecular preservation. Additionally, exploring the long-term stability of AgNPs-preserved tissues and their compatibility with advanced molecular techniques, such as next-generation sequencing, could further validate their utility. By addressing these challenges, AgNPs could offer a safer, more effective alternative to formalin, mitigating health and environmental risks while enhancing the quality of tissue preservation for research and clinical applications.

### Limitations Section

This study provides significant insights into the potential of AgNPs as a tissue preservative, but several limitations warrant consideration. A primary limitation was using a single operational concentration of AgNPs (50 µg/mL), selected based on preliminary experiments and literature indicating effective antimicrobial activity and low toxicity at this level [13]. While this concentration excelled in preserving DNA, RNA, and protein integrity, it resulted in suboptimal preservation of tissue morphology compared to formalin, as evidenced by pyknotic nuclei, cell shrinkage, and poor cytologic features in histological examinations (Section 2.7). Testing a range of AgNsP concentrations (e.g., 10, 25, 75, and 100 µg/mL) could optimize preservative efficacy, particularly for improving tissue morphology by balancing antimicrobial activity, enzyme inactivation, and tissue penetration. Additionally, this study evaluated preservation over a 72-h period, limiting insights into the long-term stability of AgNPs-preserved tissues. The compatibility of AgNPs-preserved tissues with advanced molecular techniques, such as next-generation sequencing, was not assessed, representing another constraint. Future studies addressing these limitations, including concentration optimization, long-term preservation assessments, and compatibility testing with advanced techniques, will enhance AgNPs’ applicability as a formalin alternative.

## 4. Materials and Methods

### 4.1. Synthesis and Characterization of AgNPs

Silver nanoparticles were synthesized via a chemical reduction method utilizing an aqueous solution of silver nitrate (AgNO_3_) as the precursor for silver ions (Ag⁺) [23]. The reduction of Ag⁺ to metallic silver (Ag^0^) was accomplished by trisodium citrate (Na_3_C_6_H_5_O_7_), which functioned as both a reducing and capping agent [18]. Briefly, 1 mM of AgNO_3_ solution was prepared in deionized water (DW) and heated to 95 °C, following which Na_3_C_6_H_5_O_7_ was added. This solution was stirred vigorously for 7 min. The resulting nanoparticles were collected by ultracentrifugation at 27,000× *g* for 15 min using a Beckman J2-MC/JA-20 centrifuge. The supernatant was decanted, replaced with DW, and then centrifuged three times at 31,000× *g* for 20 min using a Beckman L-80/NVT-65 centrifuge. The size and concentration were measured using a Malvern ZEN3600 Zetasizer Nano series (Malvern Instruments Inc., Westborough, MA, USA).

### 4.2. Preparation of Tissue Samples

Tissue samples, specifically liver, heart, and kidney, were obtained from adult BALB/c mice that had reached their natural endpoints. The current study was approved by the institutional research and ethics committee Ref No: E9-PI-11-24. Following CO_2_ euthanasia, in accordance with institutional ethical guidelines, liver, heart, and kidney tissues were extracted and weighed to ensure uniformity. One set of samples was preserved in 10% neutral buffered formalin (10% NBF) and the other in AgNPs solution (50 ug/mL) for up to 72 h. Tissues were also preserved in saline and used as a control at 0 h. DNA, RNA, and proteins were retrieved from all tissues under different treatment conditions at 24 h intervals, and the efficiency of each preservative was compared in terms of quantity and quality and for histological examination.

### 4.3. Nucleic Acid Extraction

#### 4.3.1. DNA Extraction

Genomic DNA was extracted from the samples using the Qiagen DNA Mini Kit (catalog number 51304), according to the manufacturer’s instructions [24]. Initially, samples were homogenized in 180 µL buffer ATL, followed by the addition of 20 µL proteinase K, and incubated at 56 °C for 30 min to facilitate cell lysis. Subsequently, 200 µL of buffer AL was added, and the mixture was vortexed before the addition of 600 µL of ethanol (96–100%). The resulting solution was transferred to a mini-spin column and centrifuged at 8000 rpm for 1 min. The column was washed with 500 µL buffer AW1 and buffer AW2, and DNA was eluted with 100 µL buffer AE. Extracted DNA was stored at −20 °C for further analysis.

#### 4.3.2. RNA Extraction

Total RNA was isolated using TRIzol reagent (catalog number 15596026, Thermo Fisher Scientific Inc.) following the manufacturer’s protocol [25]. The samples were initially homogenized in 1 mL TRIzol reagent. After 5 min of incubation at room temperature, 200 µL of chloroform was added, and the mixture was shaken vigorously for 15 s before incubating for 2–3 min. The mixture was then centrifuged at 12,000 rpm for 15 min at 4 °C, resulting in clear separation of the aqueous phase containing RNA. The aqueous phase was carefully transferred to a new tube, and RNA was precipitated by adding 500 µL of isopropanol. After a 10-min incubation at room temperature, the samples were centrifuged at 12,000 rpm for 10 min at 4 °C. The RNA pellet was washed with 75% ethanol, air-dried, and then dissolved in 30 µL of RNase-free water. The extracted RNA was stored at −80 °C for future assays.

#### 4.3.3. Quality Assessment

RNA and DNA quality and quantity were assessed using a spectrophotometer, which measures the amount of UV light absorbed by a sample. The ratio of absorbance at 260 nm and 280 nm is used to assess the purity of DNA and RNA. A ratio of ~1.8 is generally accepted as “pure” for DNA; a ratio of ~2.0 is generally accepted as “pure” for RNA. If the A260/A280 ratio is appreciably lower in either case, it may indicate the presence of protein, phenol, or other contaminants that absorb strongly at or near 280 nm [26]. Moreover, DNA purity was assessed via agarose gel electrophoresis, employing a 0.6% agarose gel prepared and solidified with wells for sample loading. Following mixture with a loading dye, DNA samples were introduced into these wells. Electrophoresis was performed in a buffer-filled chamber under an electric current, facilitating the separation of negatively charged DNA fragments based on size. Subsequently, the gel was stained with ethidium bromide and visualized under UV light [27].

#### 4.3.4. cDNA Synthesis and PCR

cDNA synthesis was performed using the Applied Biosystems High-Capacity cDNA Reverse Transcription Kit (catalog number 4368814-Thermo Fisher Scientific Inc., Waltham, MA, USA). In a PCR tube, 10 µL of 2× Reverse Transcription Master Mix was combined with 10 µL of the RNA sample (1 µg) and RNase-free water to achieve a total volume of 20 µL [28]. The reaction was incubated in a thermal cycler at 25 °C for 10 min, followed by incubation at 37 °C for 120 min, 85 °C for 5 min, and then held at 4 °C. After cycling, the synthesized cDNA was stored at −20 °C for subsequent PCR analysis. PCR amplification of GAPDH was conducted using SYBR Green Master Mix and an Applied Biosystems 7500 Fast Real-Time PCR System. The reaction mix consisted of 5 µL of master mix, 0.5 µL each of forward and reverse primers (5 µM) as shown in Table 1, 2 µL of cDNA template, and 2 µL of nuclease-free water [28]. The thermal cycling conditions included an initial denaturation at 95 °C for 10 min, followed by 40 cycles of denaturation at 95 °C for 15 s, annealing at 61 °C for 30 s, extension at 72 °C for 30 s, and a final extension at 72 °C for 5 min.

### 4.4. Protein Extraction and Analysis

To extract proteins from 10% NBF-fixed tissues, AgNPs, and saline, samples were collected and cut into small pieces (1–2 mm) using sterile scissors or a scalpel. The tissue pieces were placed into microcentrifuge tubes, and 200 µL of RIPA lysis buffer containing protease inhibitors was added to maintain protein integrity throughout the process. RIPA lysis buffer containing 50 mM Tris-HCl (pH 7.4), 150 mM NaCl, 1% Nonidet P-40, 0.5% sodium deoxycholate, 0.1% SDS, and protease inhibitors was thoroughly mixed with the tissue. The lysate was then incubated on ice for 30 min with occasional vortexing to enhance the lysis. After incubation, the lysate was centrifuged at 12,000× *g* for 10–15 min at 4 °C, and the supernatant containing soluble proteins was carefully collected [29]. For protein quantification, a series of BSA standards (0, 100, 200, 400, 800, and 1000 µg/mL) were prepared by diluting BSA in the same buffer used for extraction. The protein extracts were diluted in RIPA buffer (1:5, or as needed) for optimal measurement. To each standard and sample in a microplate or cuvette, 200 µL Bradford reagent was added, mixed gently, and incubated at room temperature for 5–10 min. The absorbance was measured at 595 nm using a spectrophotometer, and a standard curve was generated from the BSA standards to calculate the protein concentration in the samples. Finally, the extracted protein samples were stored at −80 °C for long-term storage or used immediately for downstream applications.

#### SDS-PAGE and Western Blot

The protein samples were mixed with the sample buffer and heated at 95 °C for 5 min. The samples were loaded into 12% gradient gel wells and subjected to electrophoresis at 120 V until the dye front reached the gel bottom. Following electrophoresis, Coomassie staining was performed, and the gels were transferred to a nitrocellulose membrane using the wet transfer method for Western blot detection. The membrane was blocked with 5% non-fat milk for 1 h at room temperature, followed by overnight incubation with GAPDH antibody (1:500) at 4 °C. After washing, the membrane was incubated with a secondary anti-mouse antibody (1:1000) for 1 h at room temperature. Detection was performed using WesternSure PREMIUM (Catalog No.50-489-552—LI COR BIOTECH LLC 92695000-Thermo Fisher Scientific Inc.) [30]. Chemiluminescent Substrate reagents were used to visualize protein bands.

### 4.5. Tissue Processing and Staining

The mouse liver, kidney, and heart specimens were fixed in 10% NBF or AgNPs solution for 24 h, 48 h, and 72 h at room temperature (18–23 °C). The tissues were then processed as usual. In brief, the tissues were dehydrated through an ethanol series, cleared with xylene, and infiltrated with paraffin. The tissues were then embedded in paraffin and sectioned at 4 μm thickness using a microtome. Hematoxylin and eosin (H&E) staining was performed for each tissue sample according to standard protocol to examine the histomorphologic features. The stained sections were digitized and examined utilizing (Aperio LV1, Leica Biosystems, United States) at 40× objective magnification. The tissues were then scored based on previously published histopathological criteria, which are nuclear staining, cytoplasmic staining, tissue morphology, clarity of staining, and uniformity of staining [31]. Each of the variables was given a score of 1 if it was acceptable/present and a score of zero if it was unacceptable/absent. When the total score was ≤2, it was graded as inadequate fixation, and when the total score was 3–5, it was graded as adequate fixation.

### 4.6. Statistical Analysis

Statistical analysis was performed using the Prism 10 software (GraphPad Software, San Diego, CA, USA). The data are presented as mean ± standard deviation (SD) for each treatment group at each time point. The statistical significance of differences between the AgNPs and formalin treatments was assessed using an unpaired, two-tailed Student’s *t*-test. A *p*-value of less than 0.05 (*p* < 0.05) was considered to indicate statistical significance.

## 5. Conclusions

Silver nanoparticles offer a promising alternative to formalin fixation, particularly due to their demonstrated efficacy in preserving DNA, RNA, and protein quantity and integrity. However, their limitations in morphological preservation pose a challenge, necessitating the need for further research and optimization. Given the established health and environmental concerns associated with formalin, pursuing alternative methods such as AgNPs is warranted.

## Figures and Tables

**Table 1:** The primer sequences.

**Gene Name**	**Product Size**	**Primers**
GAPDH	120 bp	F- AGG TCG GTG TGA ACC GAT TTG R- TGT AGA CCA TGT AGT TGA GGT CA

## Data Availability

Data supporting the findings of this study are available on request from the corresponding author.

## Abbreviations

The following abbreviations are used in this manuscript:

AgNPs	Silver nanoparticles
NBF	Neutral buffered formalin
DLS	Dynamic Light Scattering
DNA	Deoxyribonucleic acid
RNA	Ribonucleic acid
GAPDH	Glyceraldehyde-3-phosphate dehydrogenase
SDS-PAGE	Sodium Dodecyl Sulfate-Polyacrylamide Gel Electrophoresis

## Appendix A

**Table A1:** Histopathologic scoring of the liver, kidney, and heart specimens after fixation with 10% NBF or AgNP for 24 h, 48 h, and 72 h.

**Preservative**	**Organ**	**Time (Hours)**	**Nuclear Staining**	**Cytoplasmic Staining**	**Tissue Morphology**	**Clarity of Staining**	**Uniformity of Staining**	**Total Score**	**Interpretation**
10% NBF	Liver	24	1	1	1	1	1	5	Adequate
		48	1	1	1	1	1	5	Adequate
		72	1	1	1	1	1	5	Adequate
	Kidney	24	1	1	1	1	1	5	Adequate
		48	1	1	1	1	1	5	Adequate
		72	1	1	1	1	1	5	Adequate
	Heart	24	1	1	1	1	1	5	Adequate
		48	1	1	1	1	1	5	Adequate
		72	1	1	1	1	1	5	Adequate
AgNPs solution	Liver	24	0	0	0	1	1	2	Inadequate
		48	0	0	0	1	1	2	Inadequate
		72	0	0	0	1	1	2	Inadequate
	Kidney	24	0	0	0	0	1	1	Inadequate
		48	0	0	0	0	1	1	Inadequate
		72	0	0	0	0	1	1	Inadequate
	Heart	24	0	0	0	1	1	2	Inadequate
		48	0	0	0	1	1	2	Inadequate
		72	0	0	0	1	1	2	Inadequate
